# Supplementary material for: Sport Participation and Gender Differences in Dietary Preferences: A Cross-Sectional Study in Italian Adults
Source: Sports (Basel). 2025 Aug 6;13(8):258. doi: 10.3390/sports13080258 (PMC12390595; doi:10.3390/sports13080258)
Supplement: Supplementary file 1 [file sports-13-00258-s001.zip › sports-3718105-supplementary.pdf]

Supplementary Table S1. Gender differences in food preferences in the overall population.

Food Item	% Yes in Females	% Yes in Males	p-value
White Meat	83,8	89,7	0.002419
Vegetable drinks (eg soy milk)	36,5	31,3	0.002783
Low-fat white yogurt	55,3	58,2	0.007652
Tofu	20,5	19,1	0.01174
Dark chocolate (at least 70%)	78	72,4	0.01566
Legumes	85,2	86,1	0.02124
Eggs	85	88,4	0.02627
Cereals (eg spelled. barley)	77,7	73,2	0.02784
Fish	81,5	84	0.1951
Nuts	85,7	83,8	0.3787
Cow's milk	61,5	61,4	0.5411
Smoker	23,6	22,5	0.5536
Fruits	88,8	88,4	0.8267
Fresh cheeses	83,5	82,7	0.9129

Processed Meat (es prosciutto)	84,2	90,9	1.166e-07
Red Meat	74,3	87,5	1.445e-11
Cooked vegetables	92,3	84,5	1.712e-12
Raw vegetables	69,1	62,3	2.123e-05
General Meat	81,1	91,9	2.901e-07
Whole grains food	81,3	73,1	7.467e-09

The table shows the proportion of “Yes” responses for each food item, stratified by gender, in the full study population. P-values were calculated using chi-square tests, considering only valid responses (“Yes”, “No”, “Reluctantly”, “Don’t know”). Significant differences were found for several foods, including cooked vegetables, red meat, and whole grains, with women showing a greater preference for plant-based options and men for meat-based items.

Figure supplementary S1 Full comparison of food preferences by type of sport and gender

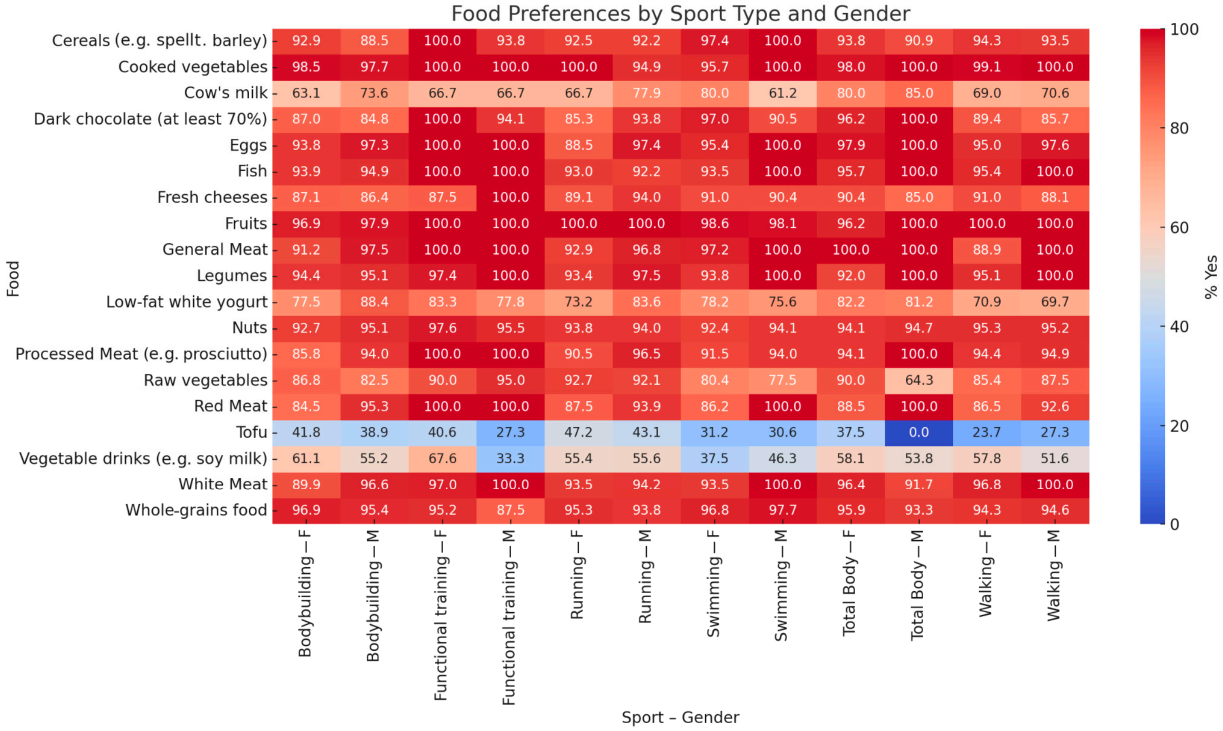

This heatmap presents the percentage of participants who reported ‘Yes’ to consuming each of the 19 foods, stratified by the six most common sport types (bodybuilding, running, walking, swimming, total body and pilates) and gender (F = female, M = male). The data include the combined responses of both primary and secondary sport types. While most sport × sex combinations showed similar dietary patterns, statistical analysis revealed significant differences between the sexes only within the bodybuilding group: processed meat ($p = 0.0018$), low-fat yoghurt ($p = 0.0023$), red meat ($p = 0.0026$), cow's milk ($p = 0.0150$) and white meat ($p = 0.0317$). All other differences were not statistically significant ($p > 0.05$).

Supplementary Table S2. Association between food preferences and sports participation for all 19 food items (multivariable logistic regression).

Food	β	SE	CI 2.5%	CI 97.5%	p-value	OR	OR 2.5%	OR 97.5%
Cow's milk	-0.21	0.10	-0.40	-0.02	0.0307	0.81	0.67	0.98
Vegetable drinks (e.g. soy milk)	0.57	0.10	0.38	0.75	0.0000	1.76	1.46	2.13
Low-fat white yogurt	0.44	0.11	0.23	0.65	0.0000	1.55	1.26	1.91
Fresh cheeses	-0.02	0.14	-0.29	0.26	0.9054	0.98	0.74	1.30
General Meat	0.42	0.27	-0.10	0.94	0.1160	1.52	0.90	2.56
White Meat	-0.16	0.22	-0.59	0.27	0.4692	0.85	0.56	1.31
Red Meat	-0.15	0.18	-0.51	0.22	0.4286	0.86	0.60	1.24
Processed Meat (e.g. prosciutto)	-0.09	0.15	-0.39	0.21	0.5685	0.92	0.68	1.24
Fish	0.40	0.18	0.05	0.76	0.0264	1.50	1.05	2.13

Eggs	0.03	0.21	-0.37	0.44	0.8688	1.03	0.69	1.55
Legumes	-0.16	0.21	-0.58	0.26	0.4538	0.85	0.56	1.29
Cooked vegetables	0.93	0.30	0.34	1.52	0.0021	2.53	1.40	4.57
Raw vegetables	0.26	0.13	0.01	0.51	0.0389	1.30	1.01	1.66
Fruits	0.68	0.27	0.15	1.22	0.0128	1.98	1.16	3.40
Cereals (e.g. spelt. barley)	0.21	0.18	-0.14	0.56	0.2417	1.23	0.87	1.75
Whole-grain foods	0.67	0.18	0.32	1.02	0.0002	1.96	1.38	2.78
Nuts	0.30	0.17	-0.04	0.64	0.0889	1.34	0.96	1.89
Tofu	0.50	0.11	0.28	0.71	0.0000	1.64	1.33	2.03
Dark chocolate (at least 70%)	0.46	0.16	0.13	0.78	0.0055	1.58	1.14	2.18

Supplementary Table S2. Multivariable logistic regression models evaluating the association between sports participation (dependent variable, Yes/No) and each food preference (independent variable, Yes/No), adjusted for age, gender, and smoking status. For each food, the table reports the regression coefficient (β), standard error (SE), 95% confidence interval (CI), odds ratio (OR), 95% CI for OR, and p-value. All 19 food preferences included. Statistically significant

results ($p < 0.05$) are highlighted in bold. All p-values are shown in full. Analyses include only participants with complete data for each specific food item and all covariates.

Supplementary Table S3. Associations between gender and individual food preferences among sport participants

Food item	β	SE	95% CI (β)	OR	95% CI (OR)	p-value
Cow's milk	0.20	0.11	-0.00, 0.41	1.23	1.00, 1.51	0.053
Vegetable drinks (e.g. soy milk)	-0.28	0.11	-0.49, -0.07	0.76	0.61, 0.93	0.008
Low-fat white yogurt	0.12	0.11	-0.09, 0.32	1.12	0.91, 1.38	0.274
Fresh cheeses	-0.08	0.14	-0.35, 0.19	0.92	0.71, 1.21	0.558
General Meat	1.27	0.29	0.69, 1.84	3.54	2.00, 6.28	<0.001
White Meat	0.67	0.22	0.24, 1.09	1.95	1.27, 2.98	0.002
Red Meat	1.15	0.20	0.77, 1.54	3.17	2.15, 4.65	<0.001
Processed Meat	0.72	0.17	0.40, 1.05	2.06	1.49, 2.86	<0.001
Fish	0.27	0.14	-0.01, 0.54	1.31	0.99, 1.72	0.058
Eggs	0.63	0.16	0.31, 0.95	1.87	1.36, 2.58	<0.001

Legumes	0.16	0.17	-0.17, 0.48	1.17	0.84, 1.61	0.341
Cooked vegetables	0.04	0.23	-0.42, 0.50	1.04	0.66, 1.65	0.868
Raw vegetables	0.15	0.12	-0.09, 0.39	1.16	0.91, 1.48	0.218
Fruits	0.09	0.13	-0.17, 0.35	1.09	0.85, 1.42	0.489
Cereals (e.g. spelt. barley)	0.05	0.13	-0.21, 0.30	1.05	0.81, 1.35	0.702
Whole- grain foods	0.03	0.13	-0.23, 0.28	1.03	0.80, 1.33	0.831
Nuts	0.06	0.13	-0.19, 0.31	1.07	0.83, 1.36	0.642
Tofu	0.16	0.13	-0.09, 0.41	1.17	0.92, 1.51	0.206
Dark chocolate (≥70%)	0.09	0.13	-0.17, 0.36	1.10	0.84, 1.43	0.480

Supplementary Table S3. Multivariable logistic regression models evaluating the association between gender (reference: female) and each food preference (dependent variable, Yes/No) among sport participants, adjusted for age and smoking status. For each food, the table reports the regression coefficient (β), standard error (SE), 95% confidence interval (CI), odds ratio (OR), 95% CI for OR, and p-value. All 19 food preferences included. Statistically significant results ($p < 0.05$) are highlighted in bold. Analyses include only participants with complete data for each specific food item and all covariates.
